# Lung mechanics showing sex‐based differences and circadian time‐of‐day response to bleomycin‐induced lung injury in mice

**DOI:** 10.14814/phy2.15828

**Published:** 2023-10-05

**Authors:** Chandrashekhar Prasad, Santhosh Kumar Duraisamy, Isaac Kirubakaran Sundar

**Affiliations:** ^1^ Division of Pulmonary Critical Care and Sleep Medicine, Department of Internal Medicine University of Kansas Medical Center Kansas City Kansas USA

**Keywords:** bleomycin, circadian clock, lung function, pulmonary fibrosis, static compliance

## Abstract

Idiopathic pulmonary fibrosis (IPF) is a progressive disease that impairs lung mechanical properties due to dysregulated extracellular matrix remodeling. Lung function assessment is an important physiological endpoint in the mouse model of pulmonary fibrosis (PF) that has gained a broader scientific acceptance in the field. IPF pathophysiology shows sex‐based differences, disproportionately affecting more men compared to women. Prior reports suggest that the circadian clock is perturbed during the pathogenesis of PF. We have comprehensively assessed the sex‐based differences and time‐of‐day response (at Zeitgeber time: ZT0/6:00 a.m. or ZT12/6 p.m.) in lung mechanics among sham (control) versus bleomycin (BLM)‐challenged female and male (C57BL/6: WT) mice using Flexi‐vent. BLM challenge altered lung function significantly in males in both total lung (reduced dynamic compliance, and increased resistance and elastance) as well as lung tissue‐specific parameters (increased tissue elastance and tissue damping) but less pronounced in females. BLM‐challenged mice showed a time‐of‐day response in lung function at ZT0 versus ZT12, which was pronounced in the ZT0 BLM group. Overall, these findings provide a comprehensive analysis of altered lung function in female and male mice and the time‐of‐day difference in lung function parameters following BLM‐induced lung fibrosis.

## Graphical abstract

Bleomycin challenge shows sex‐based differences and time‐of‐day response in pulmonary function (lung and tissue parameters) in vivo.

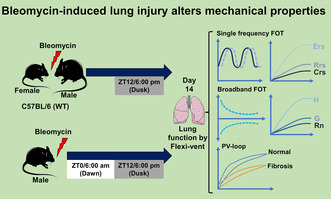

## 1 INTRODUCTION

Idiopathic pulmonary fibrosis (IPF) is an interstitial lung disease characterized by increased deposition of fibrotic tissue and aberrant inflammatory response that progressively damages the lung resulting in death due to respiratory failure (Lederer & Martinez, 2018; Wijsenbeek & Cottin, 2020). The underlying cause that triggers the development of IPF remains unknown but perhaps genetically susceptible individuals with repeated lung injury due to smoking or exposure to pollutants are predisposed to developing IPF. Therefore, a preclinical mouse model that can translate clinically relevant pathophysiological phenotypes is warranted. Bleomycin (BLM)‐induced pulmonary fibrosis (PF) in mice is among the most widely utilized translationally relevant models to study the pathobiology of PF (Jenkins et al., 2017; Moore & Hogaboam, 2008; Tashiro et al., 2017).

Biological sex and sex‐related hormones are vital to normal lung development, physiology, and diseases (Carey et al., 2007; Kawano‐Dourado et al., 2021; Sese et al., 2020). BLM‐induced PF in adult female mice shows less responsiveness to alterations in lung mechanics compared to male mice (Redente et al., 2011; Voltz et al., 2008). BLM challenge in aged male mice showed a higher incidence of mortality compared to the aged female (Redente et al., 2011). However, silica‐induced PF showed increased cellular infiltration in females versus males, which was in contrast to higher lung total hydroxyproline content in males versus females (Brass et al., 2010). Hence, the sex‐based difference in lung pathobiology exists among different models of PF.

The lung is a highly dynamic circadian organ whose response to environmental intervention is tightly regulated by the circadian clock (Giri et al., 2022). The circadian rhythm in mammals is a near 24‐h oscillation of behavioral and physiological rhythms such as the sleep–wake cycle, body temperature, feeding time, and hormone release. The circadian timing system refers to the network of transcriptional‐translational feedback loops comprised of core clock genes, such as *Bmal1*, *Clock*, *Per1/2*, *Cry1/2*, *Rev‐erbα*, *Rorα* and other clock‐controlled genes. Recently, alterations in circadian rhythms have been implicated as a major etiological risk factor that aggravates chronic inflammatory lung diseases (Giri et al., 2022).

Lung mechanical properties are among the key physiological parameters widely utilized to functionally assess PF in murine models (LaRiviere et al., 2020; Singh et al., 2017; Vanoirbeek et al., 2010; Voltz et al., 2008). BLM‐induced lung injury affects static compliance which was more pronounced in male versus female mice, whereas the other tissue‐specific parameters have been overlooked previously (Redente et al., 2011). Here, we comprehensively evaluated altered lung mechanics in the whole lung and tissue‐specific changes following BLM‐induced PF in mice. Furthermore, prior studies demonstrated the potential role of the circadian clock in the pathophysiology of PF (Cunningham et al., 2020; Pekovic‐Vaughan et al., 2014) but currently, there are no data to support the time‐of‐day response in the lung function following BLM‐induced PF. Hence, we comprehensively studied the sex‐based differences and time‐of‐day response in lung mechanics following BLM‐induced PF.

## 2 MATERIALS AND METHODS

### 2.1 Animals

All animal studies were approved and reviewed by the Institutional Animal Care and Use Committee (IACUC) of the University of Kansas Medical Center (KUMC) (ACUP# 2020‐2575‐1). All animal experiments were conducted as per the ARRIVE guidelines.

### 2.2 Bleomycin‐induced pulmonary fibrosis

Adult female and male C57BL/6 (WT; ~4–6 months old) mice were selected to develop BLM‐induced pulmonary fibrosis. Mice were dosed with BLM (1.5 U/kg) via oropharyngeal aspiration at ZT0 or ZT12, and the lung function parameters were measured on Day 14 post‐BLM challenge at the same circadian time point (ZT0/6 a.m./dawn [resting phase] or ZT12/6 p.m./dusk [active phase]). All the mice were routinely monitored for their health status, and changes in their body weight were recorded daily for 14 days.

### 2.3 Lung mechanics measurement using flexi‐vent

The lung mechanical properties were determined using Flexi‐vent (SCIREQ) instrument as reported previously (Hwang et al., 2014). A detailed description of the Flexi‐vent single forced oscillation technique (FOT) and broadband FOT measurement was described in the online supplementary information.

### 2.4 Statistical analysis

Statistical analysis of significance between sham and BLM was performed by Student t‐test (separately in females and males; Sham vs. BLM group). However, for sex‐based comparison we compared both sham and BLM from females versus males. Similarly, for time‐of‐day response, we compared sham and BLM at ZT0 versus sham and BLM at ZT12 using two‐way ANOVA followed by Tukey's post hoc testing using GraphPad Prism 9.1. The results are shown as mean ± SEM with a *p* < 0.05 considered statistically significant.

## 3 RESULTS

### 3.1 Lung mechanics in female and male sham versus BLM

BLM challenge in mice causes extensive extracellular matrix remodeling thereby affecting lung physiological functions. Dynamic compliance (*C*_rs_) a measure of the distending capabilities, elastance (*E*_rs_) which measures elastic stiffness, and resistance (*R*_rs_) which measures the constriction level within the lung inclusive of the chest wall and the airway were initially performed. We observed reduced *C*_rs_ and increased *E*_rs_ that were statistically significant and increased *R*_rs_ though it was not significant in BLM‐challenged females versus sham (Figure S1a). Broadband FOT measurement considers the lung as a two‐compartment system, central airway, and peripheral alveolar structure, and defines the tissue level changes in the lung. Tissue elastance (*H*) measures the elastance energy stored in the lung tissue and its ability to retain its original shape after forceful deformation and quantify the alveolar stiffness. Tissue damping (*G*) measures the dissipation of input energy into heat due to internal friction and represents the resistance in airflow within the peripheral airway. Newtonian resistance (*R*_n_) measures the resistance developed in a large conducting airway. *H*, *G*, and *R*_n_ in BLM‐challenged females were increased versus sham but did not reach statistical significance (Figure S1b).

Inspiratory capacity (IC) measures the total lung IC through deep inflation. IC defines the amount of air that can be held following a normal expiration. IC remains unaffected in female mice challenged with BLM versus sham. Interestingly, the parameter *K* (the coefficient of elasticity) which represents the slope of the deflation (pressure–volume curve) was reduced but not significant in sham versus BLM‐challenged female mice. Finally, static compliance (*C*_st_), which is measured under closed chest conditions, represents the intrinsic elastic property of the respiratory system in quasi‐static conditions was slightly decreased in BLM‐challenged females versus sham (Figure S1c). BLM‐challenged males showed significantly reduced *C*_rs_ and increased *E*_rs_ and *R*_rs_ versus sham. Tissue‐specific parameters *H* and *G* were significantly increased, and *R*_n_ was significantly reduced in BLM‐challenged males versus sham. Additionally, we observed significantly reduced IC, *K*, and *C*_st_ in BLM‐challenged males versus sham (Figure S1a–c). Our findings demonstrate that the female mice show minimal pathophysiological response to BLM challenge compared to male mice.

### 3.2 Comparative analysis of lung mechanics in female and male sham versus BLM

**FIGURE 1:**
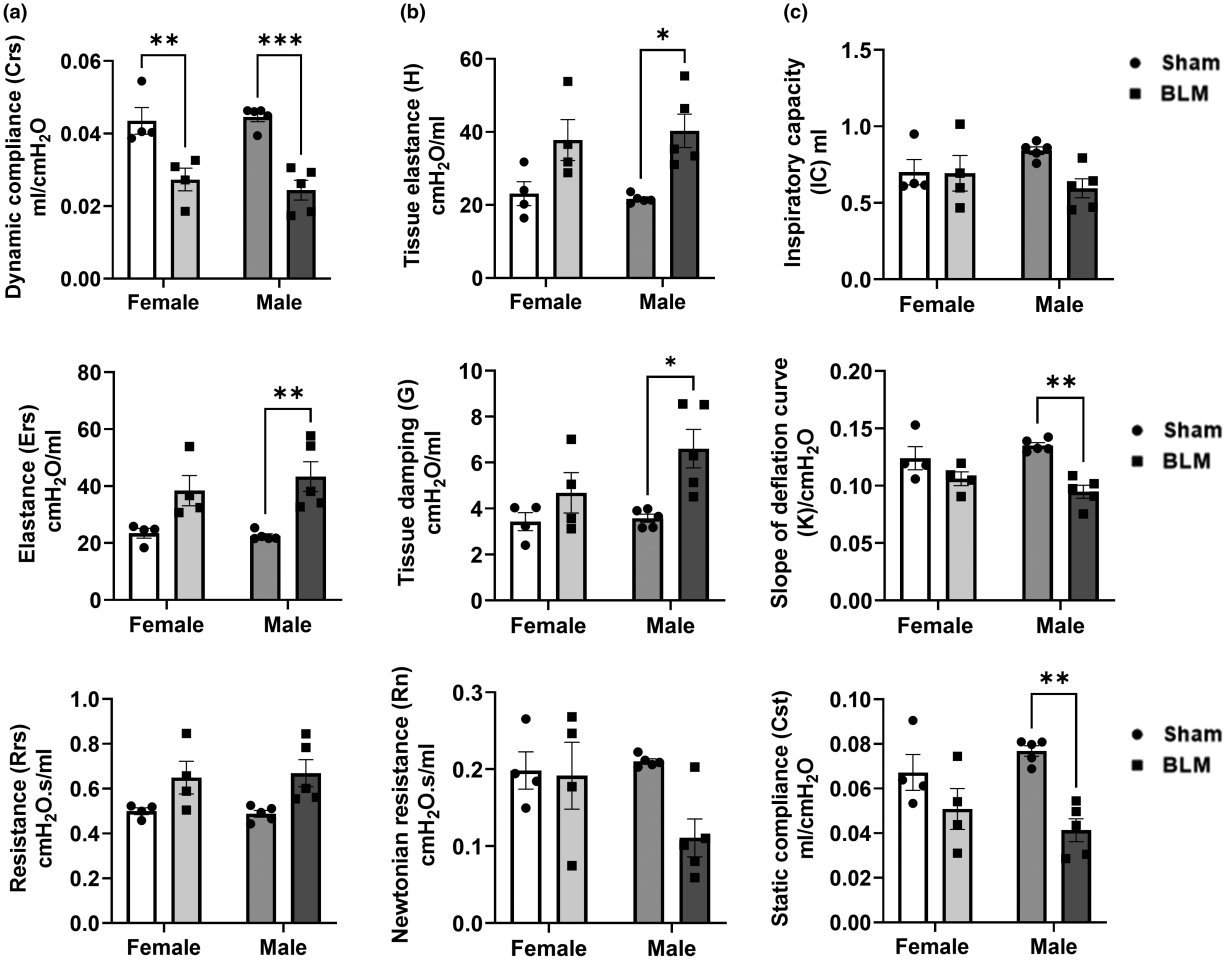
Comparative analysis showing a sex‐based difference in lung mechanics following bleomycin‐induced lung injury in mice. (a) The forced oscillation technique (FOT) was used to measure lung mechanics such as dynamic compliance (*C*_rs_), elastance (*E*_rs_), and resistance (*R*_rs_) in the lungs. (b) Mechanical properties in the lung tissue such as tissue elastance (*H*), tissue damping (*G*), and Newtonian resistance (*R*_n_) were also measured by FOT. (c) Additional parameters measured include inspiratory capacity (IC) through deep inflation, coefficient of elasticity (*K*) by the Salazar–Knowles equation, and quasi‐static compliance (*C*_st_) from PV‐curve 14 days post‐BLM and without BLM (Sham) in female and male mice. Data were shown as mean ± SEM (*n* = 4–5/group); Two‐way ANOVA followed by Tukey's multiple comparison test was used for the analysis. *p < 0.05, **p < 0.01, ***p < 0.001, compared to sham control.

We performed a grouped analysis of lung mechanics in female and male sham versus BLM‐challenged mice. We did not observe any significant difference between female versus male BLM‐challenged groups for the total lung (*C*_rs_, *E*_rs_, and *R*_rs_) as well as the lung tissue‐specific parameters (*H*, *G*, and *R*_n_) (Figure 1a,b). As expected, we observed significantly reduced *C*_rs_ in female and male BLM‐challenged groups versus respective sham. Additionally, we observed a significant increase in *E*_rs_ only in BLM‐challenged males but not in females. Lung *R*_rs_ showed an increasing trend in both females and males challenged with BLM versus sham (Figure 1a). Tissue‐specific parameters *H* and *G* were significantly increased in BLM‐challenged males but not in the females versus respective sham. However, *R*_n_ was not altered in females but reduced in males (Figure 1b). IC was reduced in BLM‐challenged males versus sham but not in females. Both *K* and *C*_st_ were significantly reduced in BLM‐challenged males versus sham but in females, both showed decreasing trends (Figure 1c). Overall, the grouped analysis revealed significant changes in the total lung and tissue‐specific parameters in BLM‐challenged males compared to females.

### 3.3 Lung mechanics in sham versus BLM at ZT0 and ZT12

We focused on males which were more responsive to BLM‐induced lung injury for time‐of‐day response at ZT0 and ZT12. BLM‐challenged mice at ZT0 and ZT12 showed significantly reduced *C*_rs_ and increased *E*_rs_ and *R*_rs_ versus sham (Figure S2a). Similarly, lung tissue‐specific changes *H* and *G* were significantly increased in BLM‐challenged mice versus sham at ZT0 and ZT12 with the exception that *R*_n_ significantly reduced in the BLM group at ZT12 but showed an increasing trend at ZT0 (Figure S2b). BLM challenge significantly reduced the IC, *K*, and *C*_st_ at ZT0 and ZT12 versus respective sham (Figure S2c).

### 3.4 Comparative analysis of lung mechanics in sham versus BLM at ZT0 and ZT12

**FIGURE 2:**
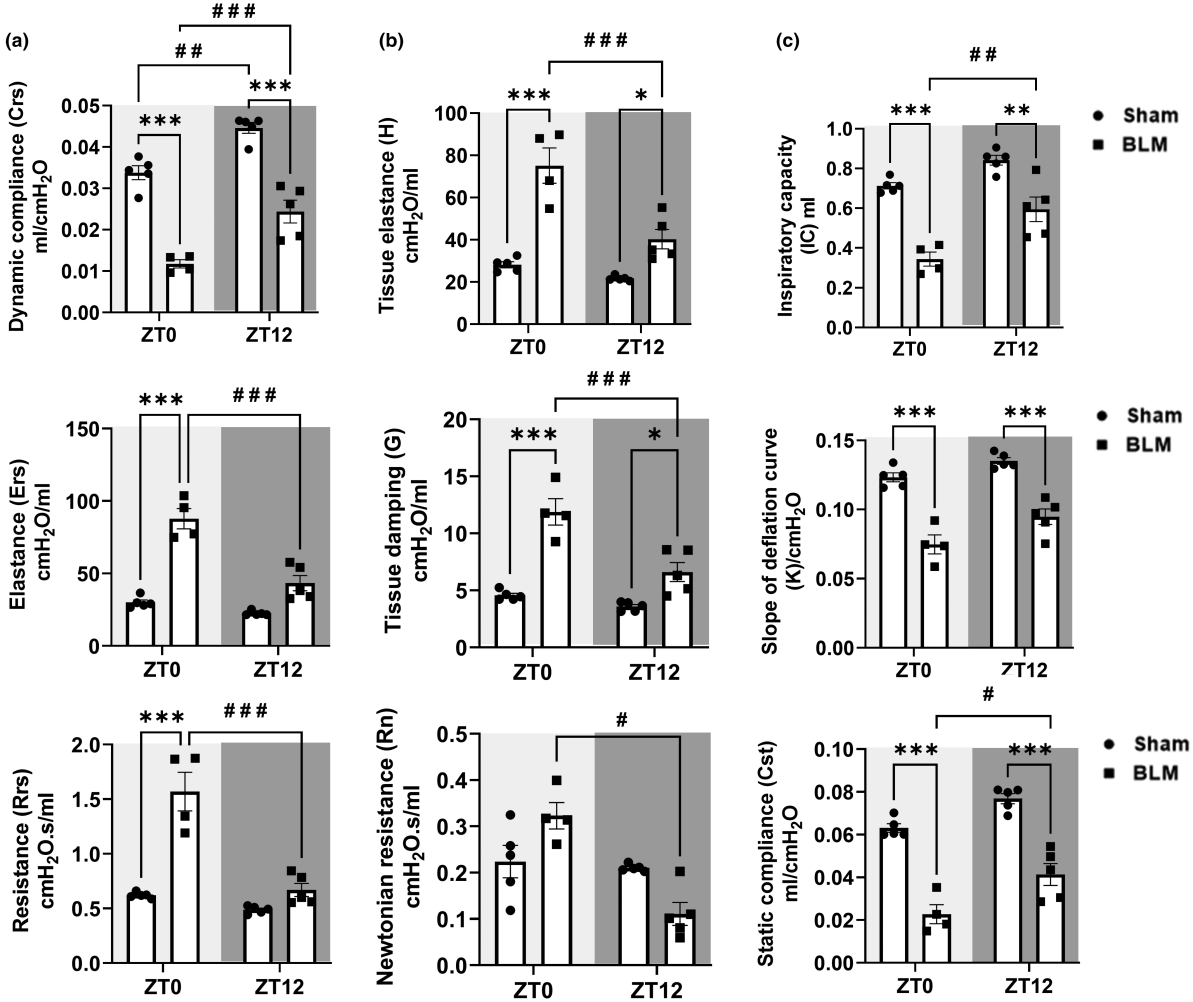
Time‐of‐day response in lung mechanics following bleomycin‐induced lung injury. (a) The forced oscillation technique (FOT) was used to measure lung mechanics such as dynamic compliance (*C*_rs_), elastance (*E*_rs_), and resistance (*R*_rs_) in the lungs. (b) Mechanical properties in the lung tissue such as tissue elastance (*H*), tissue damping (*G*), and Newtonian resistance (*R*_n_) were also measured by FOT. (c) Additional parameters measured include inspiratory capacity (IC) through deep inflation, coefficient of elasticity (*K*) by the Salazar–Knowles equation, and quasi‐static compliance (*C*_st_) from PV‐curve 14 days post‐BLM and without BLM (Sham) in male mice. Data from ZT0/resting phase (light gray) and ZT12/active phase (dark gray) are indicated. Data were shown as mean ± SEM (*n* = 4–5/group), Two‐way ANOVA followed by Tukey's multiple comparison test was used for the analysis. *p < 0.05, **p < 0.01, ***p < 0.001, compared to sham control. ^#^ *p <* 0.05, ^# #^ *p <* 0.01, ^# # #^ *p* < 0.001, compared to ZT0.

We performed a grouped analysis of lung mechanics in male sham versus BLM‐challenged mice at ZT0 and ZT12 to determine time‐of‐day response. Dynamic compliance (*C*_rs_) was the only parameter that showed a time‐of‐day response in both the sham and BLM group at ZT0 versus ZT12 (increased). BLM‐challenged mice at ZT0 and ZT12 showed significantly reduced *C*_rs_ versus respective sham. However, *E*_rs_ and *R*_rs_ were significantly increased in BLM‐challenged mice at ZT0 versus ZT12 showing a time‐of‐day response (Figure 2a). Similarly, tissue‐specific changes *H* and *G* were significantly increased in BLM‐challenged mice at both ZT0 and ZT12 versus respective sham but the ZT12 BLM showed significantly reduced level of *H* and *G* versus ZT0 BLM group showing a time‐of‐day response. The *R*_n_ was slightly increased at ZT0 BLM and decreased at ZT12 versus sham but was significantly reduced at ZT12 BLM versus ZT0 BLM group (Figure 2b). Additionally, IC, *K*, and *C*_st_ were significantly reduced at both ZT0 and ZT12 BLM groups versus respective sham. Furthermore, IC and *C*_st_ significantly increased in ZT12 BLM versus ZT0 BLM showing a time‐of‐day response (Figure 2c). Overall, most of the alterations in lung mechanics that were highly significant in BLM‐challenged mice versus sham were observed at ZT0.

### 3.5 Pressure–Volume (PV) loop measurements

**FIGURE 3:**
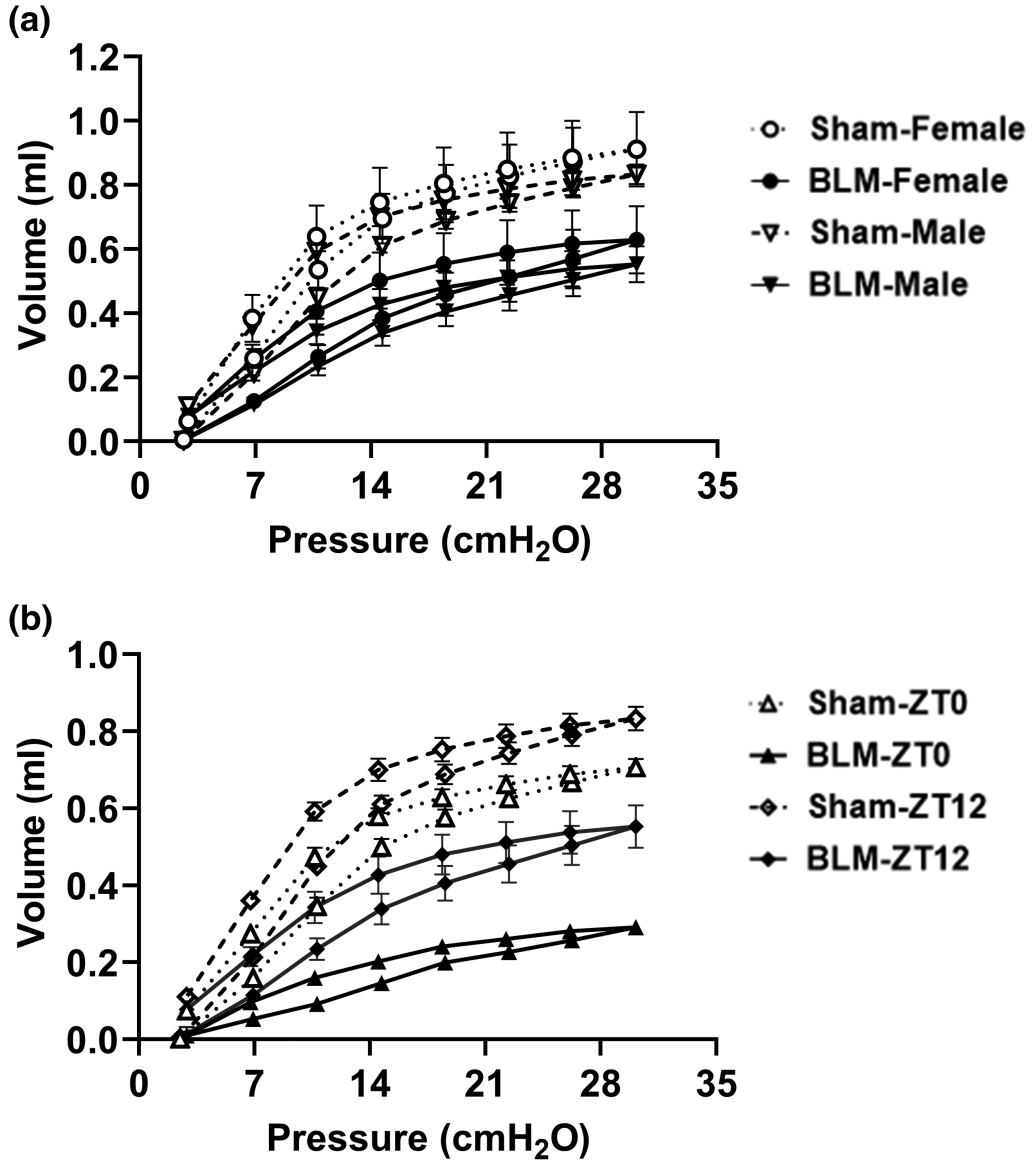
Representative PV‐curve showing a sex‐based difference and time‐of‐day response in lung mechanics following bleomycin‐induced lung injury in mice. (a) Comparison of the PV‐loop data 14 days post‐BLM and without BLM (Sham) in female and male mice at ZT12 (6:00 p.m./dusk [active phase]). (b) Comparison of the PV‐loop data 14 days post‐BLM and without BLM (Sham) in male mice at ZT0 (6:00 a.m./dawn [resting phase]) vs. ZT12 (6:00 p.m./dusk). Data were shown as mean ± SEM (*n* = 4–5/group) recorded in triplicates for each sham and BLM group.

Pressure–Volume (PV) loop measurements showed a characteristic downward shift in the curve 14 days post‐BLM challenge in both females and males, suggesting increased lung stiffness (Figure 3a and Figure S3a,b). Similarly, for time‐of‐day response experiments, PV loop measurements also showed a downward shift in the curves for the BLM group compared to the respective sham group at ZT0 and ZT12 (Figure S3c,d). Interestingly, the sham group showed a shift in the PV loop curve at ZT0 with a lower peak volume compared to the sham group at ZT12 (Figure 3b). Furthermore, the downward shift in the PV loop curve was more pronounced at ZT0 compared to ZT12 in the sham versus BLM group, indicative of higher lung stiffness 14 days post‐BLM‐induced injury (Figure 3b).

### 3.6 Lung histology—hematoxylin and eosin (H&E) and trichome staining

**FIGURE 4:**
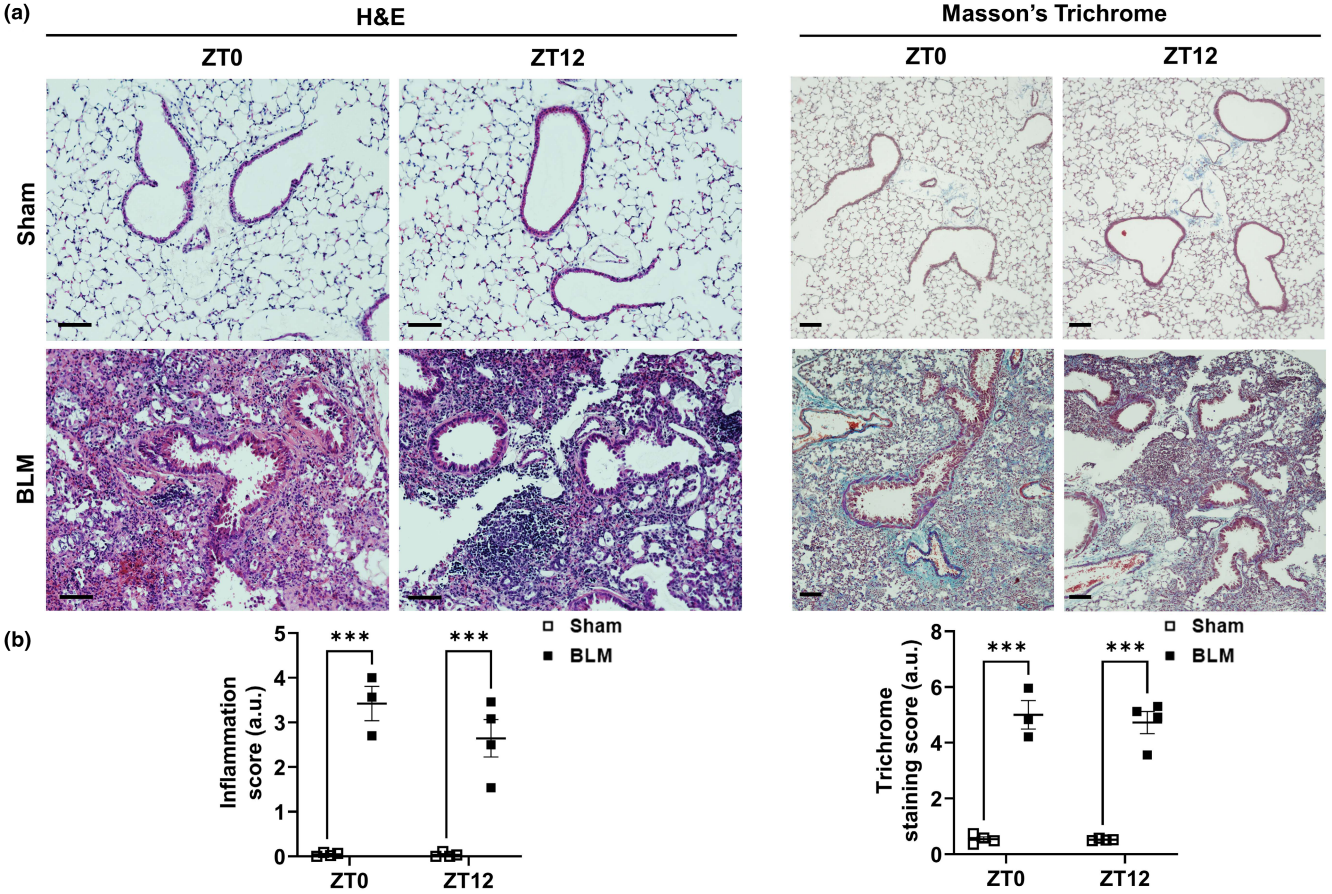
Histopathological analysis of the lung tissue following bleomycin‐induced lung injury in mice. (a) H&E‐stained lung tissue section showing inflammation, and collagen deposition (Masson's trichrome staining) 14 days post‐BLM versus sham at ZT0 (6:00 a.m./dawn [resting phase]) and ZT12 (6:00 p.m./dusk [active phase]). (b) Lung inflammation and Ashcroft's staining scores 14 days post‐BLM versus sham at ZT0 and ZT12. Data were shown as mean ± SEM (*n* = 3–5/group); Two‐way ANOVA followed by Tukey's multiple comparison test was used for the analysis. ***p < 0.001, compared to sham control. H&E‐ and Trichrome‐stained slides were imaged at 20× and 10× (scale bar 100 μm), respectively.

Hematoxylin and eosin (H&E) staining of the lung tissue section showed increased infiltration of immune cells in the peri‐bronchiolar and alveolar region of the lung in BLM versus sham at both ZT0 and ZT12. The inflammation scores were significantly increased at both ZT0 and ZT12 BLM groups versus respective sham without any time‐of‐day response (Figure 4a,b). Similarly, the Masson's trichrome staining revealed augmented collagen deposition around the peri‐bronchiolar, and alveolar regions of the lung 14 days post‐BLM injury compared to sham. Similarly, trichrome scores demonstrated increased collagen production at both ZT0 and ZT12 BLM groups versus respective sham without any time‐of‐day response (Figure 4a,b). Overall, histopathological analysis corroborates with the lung mechanics showing exaggerated inflammatory response and collagen deposition in the lung of BLM‐challenged mice.

## 4 DISCUSSION

IPF is a complex, heterogeneous progressive lung disease with unknown etiology. Preclinical models do not fully recapitulate the pathophysiology of IPF. However, it helps in the mechanistic investigation of fibrogenesis (profibrotic phenotypes) (Jenkins et al., 2017). IPF disproportionately affects the aged male (Lederer & Martinez, 2018; Wijsenbeek & Cottin, 2020), and hence, selection of the right model is a prerequisite to mimic the pathobiology of human IPF. In this study, we used adult WT mice ~4–6 months old for BLM challenge to avoid morbidity and mortality and measure changes in lung mechanics 14 days post‐lung injury in female and male mice (Stout‐Delgado et al., 2016). Researchers agree that the intratracheal instillation of BLM in male mice is widely utilized to characterize the pathophysiology of pulmonary fibrosis. In the BLM‐induced lung injury model, the change in inflammation to the fibrotic phase occurs at Day 9 and most profibrotic markers show peak expression at Day 14 (Izbicki et al., 2002; Kitzerow et al., 2022; Tan et al., 2021; Wang et al., 2015). Histopathological analysis supports prior findings as evidenced by increased lung inflammation and collagen deposition the hallmark features of pulmonary fibrosis 14 days post‐BLM in mice. Hence, we focussed to measure lung function parameters at 14 days post‐BLM challenge. Literature supports that static compliance is significantly reduced in BLM‐challenged mouse lung as early as 8–10 weeks of age (young) and adult male mice 52–54 weeks of age (middle‐aged) compared to female mice of the same age (Redente et al., 2011).

Our findings corroborated with previous reports showing a similar trend in lung function parameters (reduced *C*_rs_, increased *E*_rs_, and *R*_rs_) (LaRiviere et al., 2020; Redente et al., 2011; Vanoirbeek et al., 2010). BLM group showed pronounced changes in lung function in males when analyzed separately. Furthermore, sex‐based comparison of lung function data at 14 days post‐BLM challenge revealed significant changes in *C*_rs_, *E*_rs_, *H*, *G*, *K*, and *C*_st_ among males with an exception for *R*_rs_, *R*_n_, and IC showing trends. However, females showed significant alteration in *C*_rs_. PV loop data did not show any substantial difference (downward shift) among males versus females. Our data support BLM‐challenged male mice showed significantly altered lung function parameters compared to females in sex‐based comparison, which is in strong agreement with prior reports (LaRiviere et al., 2020; Redente et al., 2011; Vanoirbeek et al., 2010).

Idiopathic pulmonary fibrosis (IPF) disproportionally affects more men than women (Lederer & Martinez, 2018; Wijsenbeek & Cottin, 2020). There is a strong sexual dimorphism that exists in current literature that most studies only utilize male mice for the BLM‐induced lung injury/fibrosis model (Elliot et al., 2019; Hagiwara et al., 2000; Pham et al., 2022). Hence, most of the preclinical studies on pulmonary fibrosis are inclined toward using male mice instead of females. Other parameters such as profibrotic markers, hydroxyproline, collagen content, total cells, differential cell counts in BAL fluid, and TGFβ1 protein expression in lung tissues were significantly increased in adult male mice compared with female mice (Redente et al., 2011). A recent study further supports this observation that male mice are more susceptible to bleomycin‐induced pulmonary inflammation due to sex‐specific transcriptomic differences in myeloid cells (Lamichhane et al., 2022). Based on the above‐mentioned existing evidence and from our study, we utilized male mice for time‐of‐day response experiments. Another report showed that male androgen hormones might be the driving factor behind observed sex‐based differences in the pathophysiology of BLM‐induced lung fibrosis (Voltz et al., 2008). Findings from castrated male mice challenged with BLM show attenuation of *C*_st_ accompanied by reduced profibrotic response following BLM‐induced lung injury. Furthermore, 5‐α‐dihydrotestosterone treatment in female mice decreased the *C*_st_ significantly thereby enhancing their susceptibility to BLM‐induced lung injury (Voltz et al., 2008). These reports explain the causal‐effect relationship of sex‐based differences observed in adult mice challenged with BLM.

There is strong evidence in the literature that supports preclinical mouse models exposed to cigarette smoke (CS) and lipopolysaccharide showed time‐of‐day response suggesting a potential role of the circadian clock (Gibbs et al., 2014; Hwang et al., 2014). Limited reports exist about circadian regulation in pulmonary fibrosis emphasizing the need to investigate time‐of‐day response in lung function using BLM‐induced lung injury in mice. Arrhythmic clock mutant mice (*clock* ^*∆19*^) developed pulmonary fibrosis phenotype (Pekovic‐Vaughan et al., 2014). BLM‐challenged fibroblast‐specific *Rev‐erbα* knockout (KO) showed exaggerated fibrotic response associated with increased accumulation of αSMA positive myofibroblasts in the lungs (Cunningham et al., 2020).

In this report, BLM‐challenged mice showed a time‐of‐day response in lung function at ZT0 versus ZT12 circadian time points. This includes *C*_rs_, *E*_rs_, and *R*_rs_ in the whole lung, and tissue‐specific changes such as *H*, *G*, and *R*_n_. We also observed time‐of‐day response in IC and *C*_st_ in BLM‐challenged males at ZT0 versus ZT12. The current findings on baseline *E*_rs_ and *R*_rs_ observed in the sham group at ZT0 and ZT12 are comparable with those reported previously in air‐exposed WT mice from a chronic CS‐induced lung injury model (Hwang et al., 2014; Sundar et al., 2015). The exact reason behind the observed significant differences in *C*_rs_ in the sham group at ZT0 versus ZT12 and the change in Newtonian resistance (*R*_n_) in the BLM group at ZT0 versus ZT12 that followed an opposite trend remains unclear. Based on existing evidence, in healthy individuals and patients with stable obstructive lung disease the airway resistance (R_AW_) decreased during the morning followed by an increased towards noon, and late afternoon (Hruby & Butler, 1975). Additional evidence from clinical studies showed a correlation based on the shape of the PV loop and the degree of lung fibrosis in biopsies. These findings suggesting PV loop data can be used to predict the degree of fibrosis which is essential in the clinical evaluation of fibrosis progression (Headley et al., 2018; Sansores et al., 1996). Similarly, we found BLM challenge affects the distending capabilities (decreased *C*_rs_) of the lung as reflected by lower pressure–volume compared to sham at similar transpulmonary pressure. Overall, PV loop data showed a comparatively higher downward shift at ZT0 versus ZT12 following BLM injury. The above‐mentioned findings corroborate with lung function parameters that showed significantly reduced lung function at ZT0 versus ZT12 BLM group for time‐of‐day response. Existing studies from the circadian clock in lung injury models conduct exposures and functional measurements at the same time to determine time‐of‐day response (e.g., ZT0 vs. ZT12) (Durrington et al., 2020; Hwang et al., 2014; Srinivasan et al., 2023; Wang et al., 2023). Hence, we followed a similar experimental design for the BLM‐induced lung injury model to measure lung function changes post‐BLM challenge at ZT0 or ZT12. These findings strongly support how the time of exposure to environmental agents/chemicals can potentially influence the pathophysiological outcomes in preclinical models of lung injury.

### 4.1 Limitations

Our study mainly focused on utilizing female and male mice for lung function measurements in sham versus BLM‐challenged groups at the ZT12 time point. Additionally, we focused only on males for time‐of‐day response comparison analysis. It remains unclear whether the specific time of BLM challenge and/or lung function measurement is crucial [(ZT0 <‐> ZT12 and vice versa) BLM challenge and lung function measurement at the same time point or at a different time point] to determine the time‐of‐day response in physiological outcomes which may require further investigation. However, the precise mechanism underlying the observed time‐of‐day response remains unknown, necessitating further research employing using circadian clock gene‐specific knockout mice, as well as an examination of profibrotic phenotypes that drive time‐of‐day response differentially in BLM‐induced lung injury. However, it remains unclear whether the specific time of BLM challenge and/or lung function measurement is crucial to determine the time‐of‐day response in physiological outcomes which may require further investigation. Our finding supports the importance of physiological parameters such as lung mechanics in the lung injury model as a valuable tool to study disease progression and develop approaches to test novel therapeutics for the treatment of pulmonary fibrosis.

## AUTHOR CONTRIBUTIONS

Isaac Kirubakaran Sundar, Chandrashekhar Prasad, and Santhosh Kumar Duraisamy: designed the study and conducted the experiments; Isaac Kirubakaran Sundar and Chandrashekhar Prasad: primarily responsible for the experimental design, analyzed the data, critical interpretation of the data, preparation of figures, and drafting the manuscript. Isaac Kirubakaran Sundar, Chandrashekhar Prasad, and Santhosh Kumar Duraisamy: checked the content and approved the final version of the manuscript. We authors would like to acknowledge Prof. Bela Suki (Boston University) for his input on the time‐of‐day response phenotype observed in lung mechanics from the sham group.

## FUNDING INFORMATION

This work was supported in part by the National Institute of Health NIH R01HL142543 (I.K.S), *KUMC Research Institute, Inc* and the University of Kansas Medical Center, School of Medicine, Internal Medicine Start‐Up Funds (I.K.S.).

## CONFLICT OF INTEREST STATEMENT

The authors declare that they have no conflict of interest.

## 5 ETHICS STATEMENT

All experimental procedures were ethically approved by the Institutional Animal Care and Use Committee (IACUC) of the University of Kansas Medical Center (KUMC).

## Supporting information


Data S1.

Data S2.
