# Supplementary material for: Lung mechanics showing sex‐based differences and circadian time‐of‐day response to bleomycin‐induced lung injury in mice
Source: Physiol Rep. 2023 Oct 5;11(19):e15828. doi: 10.14814/phy2.15828 (PMC10555704; doi:10.14814/phy2.15828)
Supplement: Supplementary file 1 — Data S1. [file PHY2-11-e15828-s001.docx]

**Online Supplementary Information**

**Lung mechanics showing sex-based differences and circadian time-of-day response to bleomycin-induced lung injury in mice**

Chandrashekhar Prasad, Santhosh Kumar Duraisamy, and Isaac Kirubakaran Sundar*

Department of Internal Medicine, Division of Pulmonary Critical Care and Sleep Medicine,

University of Kansas Medical Center, Kansas City, Kansas, USA.

**Materials and Methods**

**Supplementary Figures S1-S3**

**Methods**

**Animals**

All animal studies were approved and reviewed by the Institutional Animal Care and Use Committee (IACUC) of the University of Kansas Medical Centre [KUMC] (ACUP# 2020-2575). C57BL/6 wild-type (WT) female and male mice were obtained from the Jackson laboratory. All animals were routinely maintained in the light-dark cycle (L:D; 12:12) with ad libitum access to food and water at the Research Support Facility (RSF) vivarium at KUMC until they reached the experimental age of ~4-6 months. All animal experiments were conducted as per the ARRIVE guidelines.

**Bleomycin-induced lung fibrosis**

Adult female and male C57BL/6 (WT) mice were selected to develop BLM-induced pulmonary fibrosis. Female mice (~4-6 months old; ~18-20 g body weight) were randomly divided into two groups (sham ZT12 and BLM ZT12 groups). Male mice (~4-6 months old; ~23-28 g body weight) were randomly assigned into four groups (sham ZT0 and BLM ZT0, sham ZT12, and BLM ZT12). The above-mentioned female and male mice were dosed with BLM (1.5 U/kg) via oropharyngeal aspiration at ZT0 or ZT12 and the lung function parameters were measured 14 days post-BLM challenge at the same circadian time point (ZT0/6 am/dawn or ZT12/6 pm/dusk). Female and male sham groups (naïve) did not receive any treatment. Lung function parameters in sham and BLM groups were measured at the same circadian time points (ZT0 or ZT12) on day 14. All the mice were routinely monitored for their health status and change in their body weight was recorded daily until 14 days.

***Lung mechanics measurement using Flexi-vent***

After 14 days post-BLM challenge mice were anesthetized using a ketamine and xylazine mixture (Dose: Ketamine hydrochloride 90 mg/kg body weight and Xylazine 10 mg/kg body weight) via intraperitoneal injection. A calibrated catheter (22 gauge and 25 mm long) was inserted in the trachea of the anesthetized mouse by making a small incision and tying it with a suture. In the meantime, the Flexi-vent (SCIREQ) instrument was set up and calibrated before loading the mice for recording lung function parameters. In brief, the Flexi-vent instrument was calibrated using FlexiWare (version 8.2) at four different stages. This includes the cylinder pressure channel and airway opening pressure channel with two-point calibration (0 mm H_2_O and 300 mm H_2_O) showing values within the expected range. Then, we proceeded with the dynamic tube calibration of the catheter in both open and closed conditions for selected perturbations such as Deep Inflation, SnapShot-150, Quick Prime-3, and PVs-P. The open and closed calibration was performed using the catheter before selected perturbation was performed in each mouse. The catheterized mouse was connected to the instrument through y-tubing using the calibrated catheter and the lung function parameters were recorded using mouse default perturbations. The single forced oscillation technique (FOT) was used to measure dynamic compliance (Crs), elastance (Ers), and dynamic resistance (Rrs) of the lung as a single compartment. Broadband FOT was used to assess the parameters such as tissue elastance (H), tissue dampening (G), and Newtonian resistance (Rn) by considering the lung as a partition system consisting of a central airway and peripheral alveolar structure. Inspiratory capacity (IC) was determined by recruiting the mice through deep inflation. The slope of the deflation curve (K) and quasi-static compliance (Cst) were extracted from PV (pressure-volume)-loop manoeuvre to define the quasi-static mechanical properties of the lung. All the lung function parameters were analyzed from the exported data files. To determine the elastic recoil of the chest wall and lung tissue during inspiration and expiration in the sham vs. BLM group were depicted as PV-loop data/graphs (see Fig. 3 and Fig. S3).

**Hematoxylin and Eosin (H&E), Trichome Staining, Modified Inflammation, and Ashcroft Scoring**

Mice were euthanized following lung function measurements and the larger left lobe was inflated with 1% low-melting agarose. The inflated lung was washed in ice-cold 1X PBS and fixed in 10% neutral buffered formalin for 48 hrs. Lung tissues were gradually dehydrated using ethanol gradation (30%, 50%, and 70%), finally processed and embedded in paraffin. Embedded lung tissues were sectioned (5 microns), and Hematoxylin & Eosin, and Masson's Trichrome staining were performed according to the manufacturer's instructions. Bleomycin-induced lung inflammation and collagen deposition in the peri-bronchiolar region (airway) were scored as described previously (1).

***Statistical Analysis***

Statistical significance was calculated between the two groups sham vs. BLM in females and males separately using unpaired Student’s *t*-test. The probability of significance compared with sham (control) was based on a two-tail *t*-test. Statistical differences in more than two groups (sham vs. BLM in Females and Males; sham vs. BLM in ZT0 and ZT12) were analyzed by two-way ANOVA using Tukey's multiple-comparison test with the GraphPad Prism 9 (La Jolla, CA). The results are shown as means ± SEM with a *P* < 0.05 considered statistically significant.

**References**

1. **Srinivasan A, Giri A, Duraisamy SK, Alsup A, Castro M, and Sundar IK**. Chronic HDM exposure shows time-of-day and sex-based differences in inflammatory response associated with lung circadian clock disruption. *iScience* 107580, 2023.


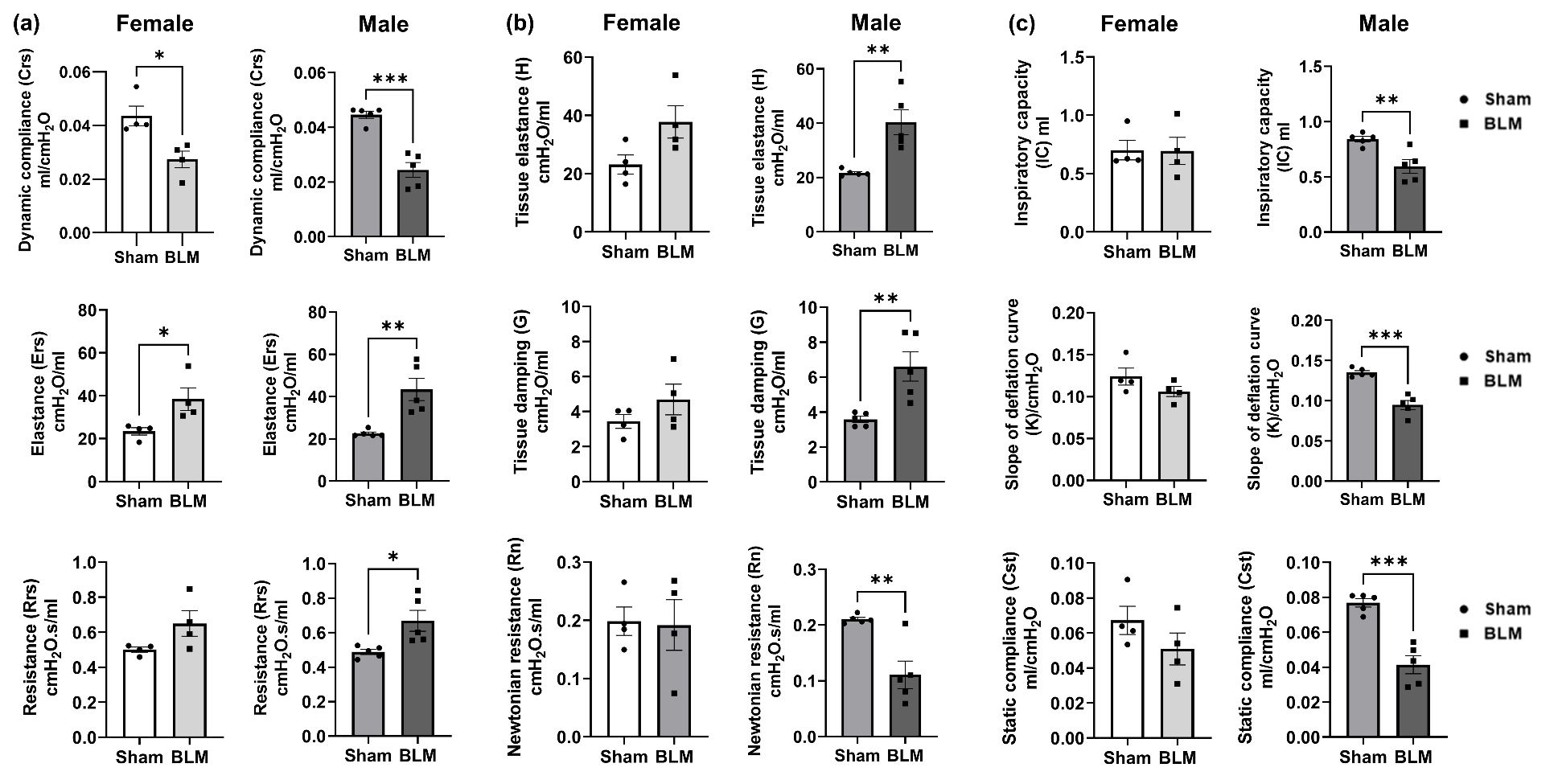


**Figure S1.** **Lung mechanics following bleomycin-induced lung injury in female and male mice.** **(a)** The forced oscillation technique (FOT) was used to measure lung mechanics such as dynamic Compliance (Crs), Elastance (Ers), and Resistance (Rrs) in the lungs. **(b)** Mechanical properties in the lung tissue such as Tissue elastance (H), Tissue dampening (G), and Newtonian resistance (Rn) were also measured by FOT. **(c)** Additional parameters measured include Inspiratory capacity (IC) through deep inflation, co-efficient of elasticity (K) by the Salazar-Knowles equation, and quasi-static compliance (Cst) from PV-curve 14 days post bleomycin (BLM) and without bleomycin (Sham) in female and male mice. Data were shown as mean ± SEM (n=4-5/group); an unpaired two-tailed t-test was used for the analysis. * *P <*0.05, ***P <*0.01, ****P* < 0.001, compared to sham control.


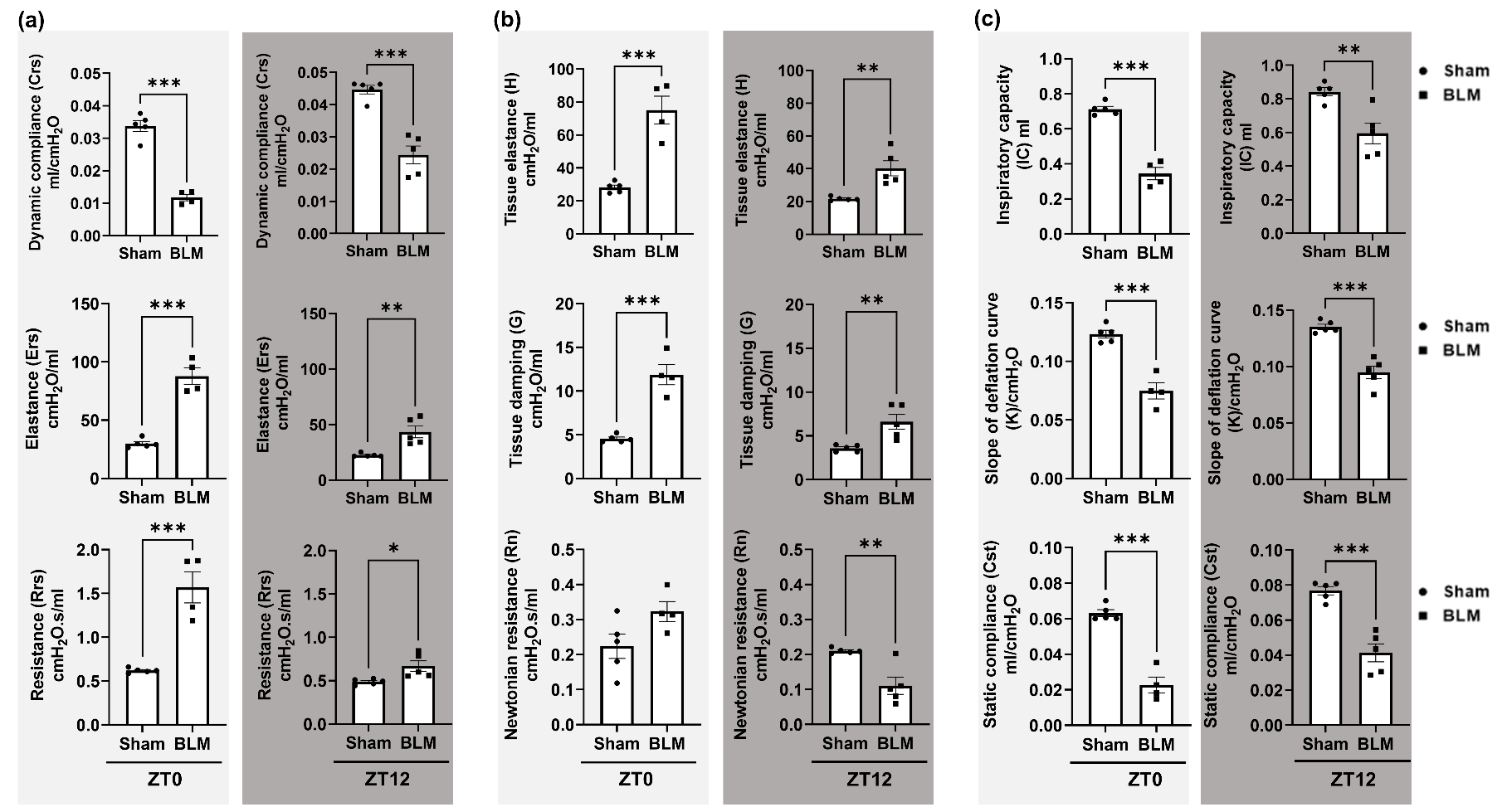


**Figure S2. Lung mechanics following bleomycin-induced lung injury at the circadian time point (ZT0/6:00 am and ZT12/6:00 pm).** **(a)** The forced oscillation technique (FOT) was used to measure lung mechanics such as dynamic Compliance (Crs), Elastance (Ers), and Resistance (Rrs) in the lungs. **(b)** Mechanical properties in the lung tissue such as Tissue elastance (H), Tissue dampening (G), and Newtonian resistance (Rn) were also measured by FOT. **(c)** Additional parameters measured include Inspiratory capacity (IC) through deep inflation, co-efficient of elasticity (K) by the Salazar-Knowles equation, and quasi-static compliance (Cst) from PV-curve 14 days post bleomycin (BLM) and without bleomycin (Sham) at ZT0 (6:00 am/dawn) and ZT12 (6:00 pm/dusk) in male C57BL/6 mice. Data were shown as mean ± SEM (n=4-5/group); an unpaired two-tailed t-test was used for the analysis. * *P <*0.05, ***P <*0.01, ****P* < 0.001, compared to sham control.


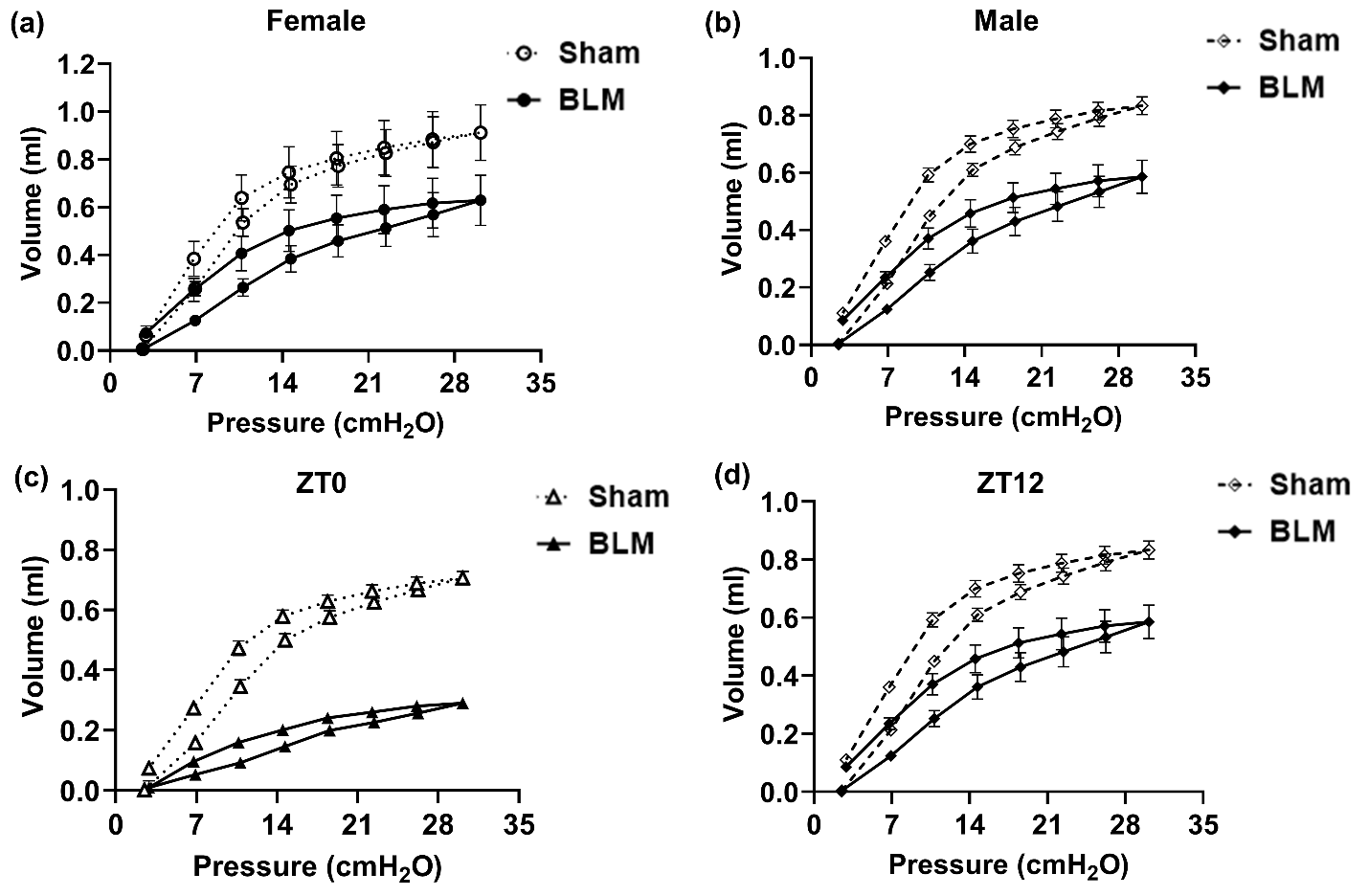


**Figure S3. Representative PV-curve showing alteration in lung mechanics following bleomycin-induced lung injury in mice.** (A-D) Comparison of the PV-loop data 14 days post- BLM and without BLM (sham) in **(a)** females at ZT12 (6:00 pm/dusk), **(b)** males at ZT12 (6:00 pm/dusk), **(c)** males at ZT0 (6:00 am/dawn) and, **(d)** males at ZT12 (6:00 pm/dusk) in C57BL/6 mice (n=4-5/group). The data are presented as mean ± SEM (n=4/group) recorded in triplicates from sham and BLM groups.
